# How can we reduce maternal mortality due to preeclampsia? The 4P rule

**DOI:** 10.61622/rbgo/2024rbgo43

**Published:** 2024-05-27

**Authors:** Henri Augusto Korkes, Ricardo Carvalho Cavalli, Leandro Gustavo De Oliveira, José Geraldo Lopes Ramos, Sérgio Hofmeister de Almeida Martins Costa, Francisco Lázaro Pereira de Sousa, Edson Vieira da Cunha, Maria Rita de Souza Mesquita, Mário Dias Corrêa, Ana Cristina Pinheiro Fernandes Araújo, Alberto Carlos Moreno Zaconeta, Carlos Henrique Esteves Freire, Carlos Eduardo Poli de Figueiredo, Edilberto Alves Pereira da Rocha, Nelson Sass, José Carlos Peraçoli, Maria Laura Costa

**Affiliations:** 1 Pontifícia Universidade Católica de São Paulo Faculty of Medicine Department of Obstetrics and Gynecology São Paulo SP Brazil Department of Obstetrics and Gynecology, Faculty of Medicine, Pontifícia Universidade Católica de São Paulo, São Paulo, SP, Brazil.; 2 Universidade de São Paulo Faculty of Medicine Department of Gynecology and Obstetrics Ribeirão Preto SP Brazil Department of Gynecology and Obstetrics, Faculty of Medicine, Universidade de São Paulo, Ribeirão Preto, SP, Brazil.; 3 Universidade Estadual Paulista "Júlio de Mesquita Filho" Botucatu Medical School Department of Gynecology and Obstetric Botucatu SP Brazil Department of Gynecology and Obstetric, Botucatu Medical School, Universidade Estadual Paulista "Júlio de Mesquita Filho", Botucatu, SP, Brazil.; 4 Universidade Federal do Rio Grande do Sul Faculty of Medicine Department of Gynecology and Obstetrics Porto Alegre RS Brazil Department of Gynecology and Obstetrics, Faculty of Medicine, Universidade Federal do Rio Grande do Sul, Porto Alegre, RS, Brazil.; 5 Centro Universitário Lusíada Department of Tocoginecology Santos SP Brazil Department of Tocoginecology, Centro Universitário Lusíada, Santos, SP, Brazil.; 6 Moinhos de Vento Hospital Porto Alegre RS Brazil Moinhos de Vento Hospital, Porto Alegre, RS, Brazil.; 7 Universidade Federal de São Paulo Paulista School of Medicine São Paulo SP Brazil Paulista School of Medicine, Universidade Federal de São Paulo, São Paulo, SP, Brazil.; 8 Universidade Federal de Minas Gerais Faculty of Medicine Department of Gynecology and Obstetrics Belo Horizonte MG Brazil Department of Gynecology and Obstetrics, Faculty of Medicine, Universidade Federal de Minas Gerais, Belo Horizonte, MG, Brazil.; 9 Universidade Federal do Rio Grande do Norte Maternidade Januário Cicco Department of Gynecology and Obstetrics Natal RN Brazil Department of Gynecology and Obstetrics, Maternidade Januário Cicco, Universidade Federal do Rio Grande do Norte, Natal, RN, Brazil.; 10 Universidade de Brasília Faculty of Medicine Department of Gynecology and Obstetrics Brasília DF Brazil Department of Gynecology and Obstetrics, Faculty of Medicine, Universidade de Brasília, Brasília, DF, Brazil.; 11 Universidade Federal do Amazonas Faculdade de Medicina Departamento de Saude Materno Infantil Manaus AM Brazil Departamento de Saude Materno Infantil, Faculdade de Medicina, Universidade Federal do Amazonas, Manaus, AM, Brazil.; 12 Pontifícia Universidade Católica do Rio Grande do Sul Departament of Nephrology and Internal Medicine Porto Alegre RS Brazil Departament of Nephrology and Internal Medicine, Pontifícia Universidade Católica do Rio Grande do Sul, Porto Alegre, RS, Brazil.; 13 Universidade Federal de Pernambuco Recife PE Brazil Universidade Federal de Pernambuco, Recife, PE, Brazil.; 14 Universidade Estadual de Campinas Department of Obstetrics and Gynecology Campinas SP Brazil Department of Obstetrics and Gynecology, Universidade Estadual de Campinas, Campinas, SP, Brazil.

**Keywords:** Gestation, Pregnancy complications, Hypertension, pregnancy-Induced, Pre-eclampsia, Aspirin, Calcium

## Abstract

In low and middle-income countries such as Brazil, most maternal deaths are related to hypertensive complications. Preeclampsia is the leading cause of maternal mortality and morbidity. Significant proportion is associated with the following factors: lack of identification of high-risk women, lack of adequate prevention, difficulty in maintaining a high-risk prenatal follow-up, delayed diagnosis, insecurity and low use of magnesium sulphate, delayed pregnancy interruption and lack of postpartum follow-up of these high-risk cases. Four major actions are proposed to minimize this alarming clinical picture and reduce the mortality rates due to preeclampsia, called the "4 P Rule" (Adequate Prevention – Vigilant Prenatal Care – Timely Delivery (Parturition) – Safe Postpartum). From this simple "rule" we can open a range of important processes and reminders that may help in the guidance of preeclampsia management.

## Introduction

Hypertensive syndromes, along with haemorrhagic and infectious syndromes, known as the "Damned Triad of Obstetrics," unfortunately are still responsible for the majority of maternal deaths worldwide.^(1,2)^ In low and middle-income countries such as Brazil, most maternal deaths are related to hypertensive complications. Preeclampsia is the leading cause of maternal mortality and morbidity.^(3)^

Significant proportion of this high maternal mortality related to hypertensive disorders is correlated with the following factors: lack of identification of high-risk women, lack of adequate prevention, difficulty in maintaining a high-risk prenatal follow-up, delayed diagnosis, insecurity and low use of magnesium sulphate, delayed pregnancy interruption and lack of postpartum follow-up of these high-risk cases.^(4)^

**Figure 1:**
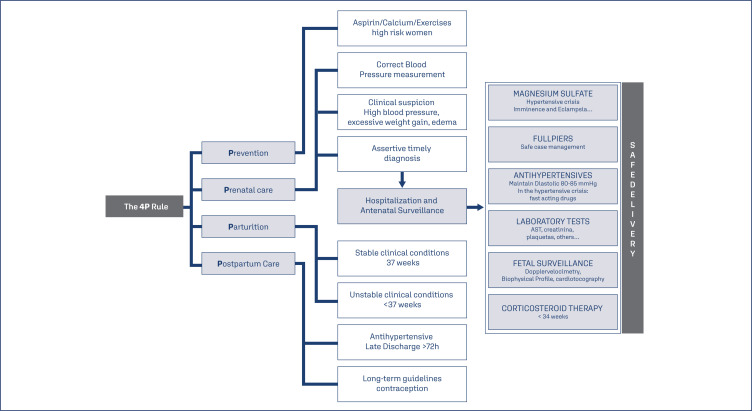
Four P rule: Obstetric mnemonic rule summarized for management of high-risk patients or those with established preeclampsia.

Four major actions are proposed to minimize this alarming clinical picture and reduce the mortality rates due to preeclampsia, called the "4 P Rule" (Adequate Prevention – Vigilant Prenatal Care – Timely Delivery (Parturition) – Safe Postpartum). From this simple "rule" we can open a range of important processes and reminders that may help in the guidance of preeclampsia management (Figure 1).

### First P: Adequate Prevention

**Figure 2:**
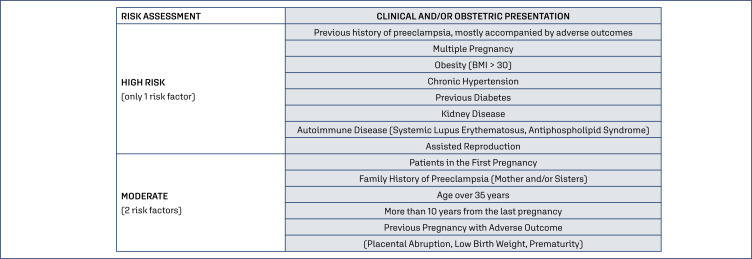
Patients at high-risk for preeclampsia, based on clinical factors.^(3)^

Source: Translated from: Peraçoli JC, Costa ML, Cavalli RC, de Oliveira LG, Korkes HA, Ramos JG, et al. Preeclampsia – Protocolo 03. Rede Brasileira de Estudos sobre Hipertensão na Gravidez; 2023. Quadro1, Fatores de risco clínicos recomendados para a identificação de gestantes com necessidade de prevenção; p. 20. [citado 2023 Mai 10]. Disponível em: https://rbehg.com.br/wp-content/uploads/2023/04/PROTOCOLO-2023.pdf.^(3)^

The best currently available evidence points towards beneficial interventions in groups at risk for the development of preeclampsia. Some of these strategies are known: use of low-dose aspirin, use of calcium for pregnant women with low calcium intake, as well as regular physical activity during pregnancy, in addition to adequate weight gain.^(3–9)^ Nevertheless, despite these being widespread recommendations in the scientific environment, in many locations there is still a low adherence to these simple and inexpensive interventions in clinical practice. The recommendations using preventive measures for preeclampsia are based on clinical risks. In this strategy, for a pregnant woman with a risk factor considered (HIGH) or two risk factors considered (MODERATE), prophylaxis should be initiated (Figure 2).

In this scenario, obesity is highlighted. It is a growing concern worldwide that increases the risk for adverse outcomes.^(10)^ Obesity causes an intense inflammatory response and may contribute to inadequate vascularization of the placenta due to high circulation of proinflammatory immune cells.^(9)^ The release of proinflammatory cytokines (TNF-α and IL-6) and other antiangiogenic factors both from the fatty tissue and ischemic placenta may result in maternal hypertension and fetal growth restriction.^(9,11)^

### Aspirin

The use of aspirin for the prevention of preeclampsia has been reported since 1979, with a major contribution from Crandon and Isherwood^(12)^ in an increasing number of publications. Its mechanism of action is still not fully understood. However, severe cases are associated to intense endothelial lesion, in addition to platelet aggregation and activation. Perhaps this may be the mechanism of action of aspirin in the prevention of preeclampsia.^(5)^

An important systematic review, published in 2007, showed the benefit of low-dose aspirin in the prevention of preeclampsia, with a risk reduction of 17% (RR, 0,83, 95% CI, 0.77–0.89).^(13)^ In 2017, ^(14)^ another meta-analysis demonstrated a slight reduction when aspirin was used beyond 16 weeks (RR, 0.81; CI, 0.66–0.99). However, there were significant benefits in the decrease in relative risk of severe preeclampsia (RR, 0.47; CI, 0.26–0.83), in addition to a reduction in fetal growth restriction (RR, 0.56; CI, 0.44–0.70), when aspirin is initiated before the 16th week.^(14)^

It is currently clear that aspirin is highly beneficial for pregnant women, not only for PE but also for reducing preterm delivery and perinatal mortality.^(5,15)^ It is known that the sooner aspirin is introduced to high-risk pregnant women, the better its protective effect will be.^(14)^ Nevertheless, in certain scenarios, especially in low-income countries where healthcare is problematic and access to prenatal care is often delayed, this preventive measure is hindered. A frequent question asked is: "Can aspirin be introduced after the 16th week? " A few studies have demonstrated a protective effect, albeit lower, in later gestational ages.^(14,16,17)^ It is currently recommended that intense effort should be made to prescribe aspirin for high-risk women, preferentially at 12 weeks. Nevertheless, in cases where this was not possible, the drug should be prescribed until 28 weeks and maintained until at least 36 week.^(3,5,18)^

The currently recommended dose of aspirin varies in the literature. While higher doses, above 100mg, could suggest better effects, some groups and clinical societies are concerned with possible side effects of the drug and recommend doses lower than 100mg.^(3,5,6)^ Other groups have suggested the use of higher doses, such as 150mg/day, with reported safety and better outcomes.^(7)^ For Brazil, the availability in the Public Health system is of 100mg, therefore supported by the national guideline.^(3,19)^

### Calcium

**Figure 3:**
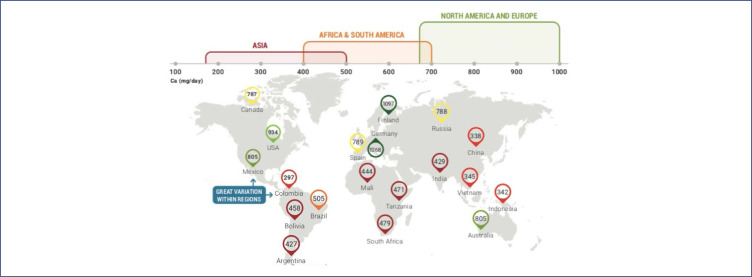
Calcium map across the world

Source: Adapted from Balk EM, Adam GP, Langberg VN, Earley A, Clark P, Ebeling PR, et al. Global dietary calcium intake among adults: a systematic review. Osteoporos Int. 2017;28(12):3315-24. doi: 10.1007/s00198-017-4230-x.^(22)^

There is a large amount of nutritional deficiencies around the world, particularly related to micronutrients.^(20,21)^ The minimum calcium intake that is adequate for a pregnant woman would be around 1000mg/day. A deficiency in calcium intake is observed mainly in countries of the southern hemisphere, at about 400 to 500mg/day (Figure 3).^(22)^

Calcium supplementation improves the availability of calcium ion systemically, reducing the need for its intracellular mobility, avoiding arteriolar smooth muscle contraction, which contributes to homeostastic pressure levels.^(23,24)^ An important Cochrane review compared two therapeutic regimens of calcium supplementation (higher or equivalent to 1 g/day and lower than 1 g/day). In this review, 27 studies (18 064 women) were included. It concluded that high-dose calcium supplementation (≥ 1 g/day) may decrease the risk of preeclampsia and preterm delivery, particularly in women with low calcium intake.^(24)^

The majority of protocols currently recommend the use of calcium supplementation for pregnant women at risk for preeclampsia and with low calcium ingestion, at doses above 1.0 gram/day.^(3,6,25)^ The World Health Organization recommends slightly higher doses, around 1.5 to 2.0 grams/day.^(26)^ All calcium presentations are better absorbed when taken in low doses (500 mg), especially during meals. Calcium citrate is different in this aspect, since it is absorbed without much interference, when not taken during meals. It is the recommended form for patients with low stomach acid, bowel inflammatory disease or absorption disorders.^(27)^

### Physical Activity

Physical activity is defined as planned, structured and repetitive body movements, aimed at improving one or more components of physical aptitude. It represents an essential part of life. During pregnancy and the postpartum period, it is understood that exercise is beneficial for the majority of patients.^(28)^

In the last decades, randomized clinical trials have demonstrated that physical activity is actually beneficial for the prevention of preeclampsia^7^. Regular exercise during pregnancy decreases the risk of gestational hypertension by 39% (OR 0,61, CI 95% 0.43, 0.85) and preeclampsia by about 41% (OR 0,59, CI 95% 0.37, 0.90).^(29,30)^

It is currently recommended that all pregnant women, mainly those at risk for diabetes and hypertension, engage in physical activity for 140 minutes per week, with moderately intense exercises such as walking, water aerobics, stationary bike and resistance training, in addition to daily chores including gardening, for example. To determine whether the activity is not too intense, a safety criterion during exercise would be that the pregnant woman should be able to talk during it. Nevertheless, in women with contraindications to physical activity, such as those already with diagnosed preeclampsia or uncontrolled arterial hypertension, this recommendation should be discouraged.^(28)^

### Second P: Vigilant Prenatal care

A thorough prenatal care, with timely interventions, based on the best evidence possible, will screen the pregnant woman for many complications during this period. Furthermore, it can help prevent diverse diseases, either by medications or even by educational guidance. In hypertensive syndromes, a careful prenatal follow-up is responsible for the clinical suspicion of preeclampsia in patients with suggestive signs and symptoms, providing the opportunity for an early diagnosis and rapid patient referral. During prenatal care, the healthcare team often composed of physicians, nurses and other health professionals will perform, in addition to other interventions, constant arterial blood pressure measurements. In this aspect, it is worth drawing attention to incorrect routine care, that is still frequently practiced.

### Arterial blood pressure measurement (ABPM)

The diagnosis of hypertension should follow correct measurement techniques, including adequate cuffs or correction tables.^(19)^ Blood pressure should be measured with the patient sitting, with feet on the floor, back and arms supported. The device is placed on the upper limb, maintaining the limb elevated at the height of the heart.^(31,32)^ The diastolic pressure (DAP) should be considered by the 5^th^ Korotkoff sound, corresponding to disappearance of the murmur.^(33)^

It is very important to highlight that the left lateral position (LLP) is used for the patient to rest, but to measure blood pressure, the patient should be sitting preferentially. The LLP position, often oriented to measure arterial pressure in pregnant women, will give false information about the real pressure and will hinder case management and should not be persued.^(31,34)^ In a similar manner, the use of cuffs that are adequate for diverse brachial circumferences is recommended. When this is not possible, the use of tables such as Maxwell's correction is recommended.^(35)^

The current use of validated electronic devices has facilitated the follow-up of these patients regarding arterial pressure measurement at home (APMH), and are great allies of the prenatal physician in pressure control, when considering validated devices.^(32,34)^

### Clinical suspicion and timely diagnosis of preeclampsia

**Figure 4:**
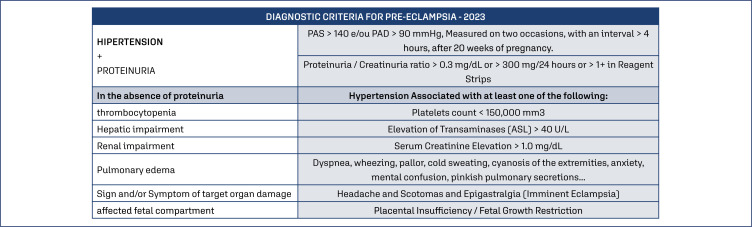
Diagnostic criteria for preeclampsia

Source: Peraçoli JC, Costa ML, Cavalli RC, de Oliveira LG, Korkes HA, Ramos JG, et al. Preeclampsia – Protocolo 03. Rede Brasileira de Estudos sobre Hipertensão na Gravidez; 2023. Quadro1, Fatores de risco clínicos recomendados para a identificação de gestantes com necessidade de prevenção; p. 20. [citado 2023 Mai 10]. Disponível em: https://rbehg.com.br/wp-content/uploads/2023/04/PROTOCOLO-2023.pdf.^(3)^

During consultations, and mainly after the 20th week, the prenatal care physician should be aware of the symptoms reported by the pregnant woman, such as general malaise, headache and body pain, nausea and vomiting, itching, visual alterations, among others. The physician should also be aware of weight gain, especially when it is above 1 kg per week, in addition to edema that may occur, usually in the hands and face. In the presence of suggestive signs and/or symptoms, particularly high arterial blood pressure, tests should be ordered for diagnosis.^(3)^ Diagnostic criteria for preeclampsia has suffered modifications throughout the years. Since the publications of the American College of Obstetricians and Gynecologists (ACOG)^(36)^ in 2013 and the International Society for the Study of Hypertension in Pregnancy (ISSHP)^(37)^ in 2014, proteinuria is not obligatory for its diagnosis. Recently in 2018,^(1)^ once again the ISSHP modified the diagnostic values for PE that are maintained until today (Figure 4).^(7)^

### Follow-up after diagnosis

Upon the diagnosis of preeclampsia, hospital admission of the pregnant woman is recommended for adequate follow-up of the mother and fetus.^(3)^

- *Considering maternal follow-up*: tests should be periodically ordered to evaluate systemic compromise. The PIERS calculator may help in this follow-up and in the definition of risk of maternal adverse events in the following 48 hours. In this context, the following tests are mandatory: Transaminases, Platelets, Creatinine, among others that are eventually necessary, to determine case severity.^(38)^ Regarding the pregnant woman, rigorous BP control, with the introduction of antihypertensive medication to maintain arterial pressure below 140 x 90mmHg and if necessary, the use of magnesium sulphate, mainly in cases of clinical or laboratory deterioration.^(3)^
- *Considering fetal follow-up:* fetal compromise is always possible in this scenario. Vitality tests such as cardiotocography, fetal biophysical profile, doppler velocimetry should be performed. Pulmonary maturation should also be part of fetal care, when below 34 weeks, and especially when considering childbirth, as well as neuroprotection with magnesium sulphate for foetuses at risk of birth before 32 weeks.

### Third P: Parturition-Timely childbirth

This is one of the great challenges that preeclampsia patients face. It is well-known that the definitive treatment for this life-threatening situation is the resolution of pregnancy. Nevertheless, some aspects need to be observed. In preeclampsia with severe features or maternal deterioration, delivery should occur, evidently following maternal stabilization. Blood pressure control, as well as magnesium sulphate infusion, for patients with eclampsia or imminent eclampsia, should be part of the initial care. Likewise, assessment of recent laboratory tests or solicitation of new tests is essential for identifying possible acute alterations. Concerning the delivery route, it is known that except in cases of evident need for a rapid birth, the patient will benefit from a vaginal delivery. Therefore, cervical preparation with subsequent labour induction should be the first option.^(3,25)^

**Figure 5:**
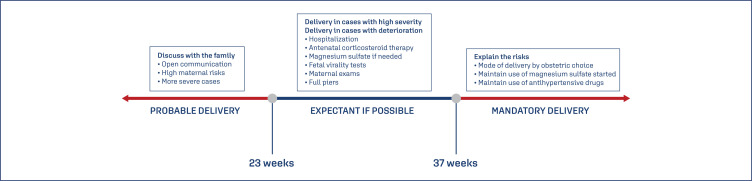
Time of preeclampsia resolution^(3)^

Source: Peraçoli JC, Costa ML, Cavalli RC, de Oliveira LG, Korkes HA, Ramos JG, et al. Preeclampsia – Protocolo 03. Rede Brasileira de Estudos sobre Hipertensão na Gravidez; 2023. Quadro1, Fatores de risco clínicos recomendados para a identificação de gestantes com necessidade de prevenção; p. 20. [citado 2023 Mai 10]. Disponível em: https://rbehg.com.br/wp-content/uploads/2023/04/PROTOCOLO-2023.pdf.^(3)^

Regarding the period of pregnancy interruption, it is known that patients that reach term (37 weeks) should be referred for childbirth.^(3,6,7,25)^ Patients with gestational age above 23 weeks and below 37 weeks, with confirmed stability of the maternal-fetal condition, a rigorous and careful follow-up is possible, taking into consideration fetal assessment. For cases below 23 weeks, due to high maternal risks and fetal pre-viability, there should be an open conversation with the parents, explaining about possible severe maternal outcomes, for deciding on the best management possible (Figure 5).^(3)^ In the context of viability, that involves diverse aspects, many related to the intensive care unit and the pediatric team, the pediatric team should participate in all decisions. Viability concepts are in constant modification, and the multidisciplinary team need to participate in this decision---the patient, family members, obstetricians and neonatologists.

### Fourth P: Safe Postpartum period

In the immediate postpartum period and days following childbirth, the medical team should remain vigilant and monitor any potential complications. Blood pressure is a major concern, therefore it is mandatory to monitor arterial blood pressure every four hours or more frequently according to specific cases. In general, antihypertensive medication should not be stopped abruptly after delivery, even if the patient has hypotension secondary to anesthetic procedures. In a similar manner, for patients that arrived at the time of delivery using magnesium sulphate, this medication should be maintained for 24 hours after childbirth.

Another relevant aspect concerns the use of unsafe medications during this period. Among these drugs, we can cite nonsteroidal antiinflammatory agentes that should be avoided. Despite the lack of robust evidence to prohibit their use in postpartum hypertensive patients, in renal compromise, for example due to major blood loss during delivery or even due to the deleterious process caused by preeclampsia, it is known that its use may worsen renal function.^(3,34)^ In a similar manner, the use of medication to suppress lactation (e.g., bromoergocriptine and cabergoline) should be avoided, since these drugs are associated with an increased risk of adverse cerebrovascular events.^(3)^

The healthcare team should be aware about clinical and/or laboratory deterioration. Therefore, laboratory reassessment is recommended at 24h and 48h after childbirth. After this period, new tests are requested, according to each case.^(39)^

It is recommended that the patient remain monitored in the hospital setting for at least 72 hours. It is known that circulatory dynamics and water reabsorption to the intravascular compartment are commonly re-established between the third and fifth days postpartum, often elevating arterial blood pressure, promoting symptoms and increasing the chance of complications.^(3)^ Thus, early hospital discharge (before day 3), increase the risk of these patients and are strongly contraindicated.

Another relevant aspect during this period is family planning, counselling patients about different and safe contraceptive methods. Making long-duration methods available, such as subdermal implants or even intrauterine devices (IUD), in addition to the performance of intrapartum tubal ligation, represent fundamental public health strategies.^(40,41)^

Furthermore, it is important to advise the woman and her family about future repercussions, explaining the severity of the case.

### Long-term follow-up of patients that developed preeclampsia

In remote times, it was believed that preeclampsia was a self-limited hypertensive disease specific to pregnancy. The cure for hypertension occurred after placental removal. After the 1990s, the first studies appeared in the literature demonstrating that hypertensive disorders of pregnancy, particularly preeclampsia, increased the risk of cardiovascular disease throughout a woman's life.^(42–45)^

In 2018, the ACOG released guidelines proposing a longitudinal action that was not only restricted to consultation in the sixth postpartum week.^(39)^ In fact, it is currently recommended that hypertensive pregnant women, delay hospital discharge for more than 72 hours. Return visit is anticipated for a maximum of 10 days.^(3)^

In 2018 and later in 2022, ISSHP also advised on a more rigid follow-up, with clear guidance about the future risk in pregnant women that had presented preeclampsia, recommending follow-up with other medical professionals for the rest of these patients’ lives.^(1,7)^

In Brazil, the Brazilian Network for Studies on Hypertension in Pregnancy (BNSHP), launched a manual for patient management, recommending the surveillance of these women throughout the years and proposing guidelines for a safer follow-up.^(44)^

## Conclusion

Finally, we should keep in mind the relevance of the sentence: "once preeclampsia, always preeclampsia" in order to draw attention to the long-term effects of this disease on the lives of these women and family and to ascertain adequate counselling about disease recurrence and possible future risks, mainly related to cardiovascular disease.
